# An uracil-linked hydroxyflavone probe for the recognition of ATP

**DOI:** 10.3762/bjoc.14.63

**Published:** 2018-04-03

**Authors:** Márton Bojtár, Péter Zoltán Janzsó-Berend, Dávid Mester, Dóra Hessz, Mihály Kállay, Miklós Kubinyi, István Bitter

**Affiliations:** 1 Department of Organic Chemistry and Technology, Budapest University of Technology and Economics, 1521 Budapest, Hungary; 2 MTA-BME Lendület Quantum Chemistry Research Group, Department of Physical Chemistry and Materials Science, Budapest University of Technology and Economics, 1521 Budapest, Hungary; 3 Institute of Materials and Environmental Chemistry, Research Center for Natural Sciences, Hungarian Academy of Sciences, P. O. Box 286, 1519 Budapest, Hungary; 4 Department of Physical Chemistry and Materials Science, Budapest University of Technology and Economics, 1521 Budapest, Hungary

**Keywords:** ATP sensing, base-pairing, fluorescent probes, 3-hydroxyflavone, nucleotide recognition

## Abstract

**Background:** Nucleotides are essential molecules in living systems due to their paramount importance in various physiological processes. In the past years, numerous attempts were made to selectively recognize and detect these analytes, especially ATP using small-molecule fluorescent chemosensors. Despite the various solutions, the selective detection of ATP is still challenging due to the structural similarity of various nucleotides. In this paper, we report the conjugation of a uracil nucleobase to the known 4’-dimethylamino-hydroxyflavone fluorophore.

**Results:** The complexation of this scaffold with ATP is already known. The complex is held together by stacking and electrostatic interactions. To achieve multi-point recognition, we designed the uracil-appended version of this probe to include complementary base-pairing interactions. The theoretical calculations revealed the availability of multiple complex structures. The synthesis was performed using click chemistry and the nucleotide recognition properties of the probe were evaluated using fluorescence spectroscopy.

**Conclusions:** The first, uracil-containing fluorescent ATP probe based on a hydroxyflavone fluorophore was synthesized and evaluated. A selective complexation with ATP was observed and a ratiometric response in the excitation spectrum.

## Introduction

Nucleotides play essential roles in various physiological processes, such as energy transportation [1], DNA synthesis [2] and cell signaling events [3]. Especially, adenosine-5’-triphosphate (ATP) is vital, since it is the main energy source in living systems [4]. The recognition and sensing of ATP has therefore paramount importance in the understanding of biological processes. Amongst the numerous solutions [5–9], fluorescent chemosensors using either indicator displacement assays [10–15], cation-based recognition units [16–20], metal-complexes [21–27] and other direct sensing systems [28–30] have significant advantages over the classical, separation-based methods. The primary difficulties in the design of an ATP chemosensor are the structural similarity of ATP to other nucleotides (i.e., to guanosine-5’-triphosphate, GTP) and the strong solvation of the chemosensor and the analyte in aqueous media, reducing the association constant of their complex, and through that the sensitivity of the sensor [31–32]. The molecular recognition of nucleotides in most chemosensors is achieved by charged recognition sites [16,18–19,33], or Zn-dipicolylamine complexes [21,23–24] attracting the negatively charged phosphate units of ATP and by π-stacking between the fluorophores of the sensors and the adenine moiety of ATP [29]. In aqueous solutions at physiological pH, the tetra-charged anionic ATP consists of a hydrophilic (phosphate and ribose) and a more hydrophobic part (adenine). The former ensures a good solubility of ATP in water and generates an electrostatic field around it while the latter is required for associations with similar planar hydrophobic molecules involved in the biochemical processes of ATP [34]. The ideal ATP probe possessing all the prerequisites to bind ATP should be sensitive to electrostatic fields in solution and in molecular assemblies as well. 3-Hydroxyflavone (HF) fluorophores, especially the highly polarizable 4’-dialkylamino subfamily exhibit strong sensitivity to electric fields generated by ions and molecules in solution. This property along the ESIPT process (excited state intramolecular proton transfer) [35] makes them ideal for ratiometric environment-sensitive probes and sensors [36–43]. Among them the 4’-dimethylamino derivative (DMHF, 4’-dimethylaminohydroxyflavone) was utilized by Pivovarenko and co-workers in ATP sensing in aqueous solution and in mitochondria [34,44]. DMHF was found to form 1:1 and 1:2 complexes with ATP. The two components were held together by π-stacking and by electrostatic interactions of the positively polarized dimethylamino group of DMHF and the negative charges of ATP. The interaction of DMHF with nucleotides and a computer aided simulation on the geometry of DMHF∙ATP complexes in vacuo was also reported. A recent study by the same authors describe further flavones with various association constants [45], however, none of them are the result of rational design.

**Figure 1:**
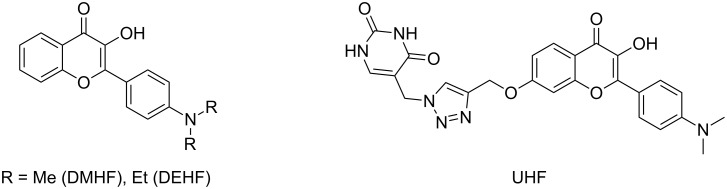
Structures of the studied hydroxyflavone derivatives.

Base pairing is a well-known phenomenon in the double helix structure of DNA since the work of Watson and Crick. It is also known that the cohesion of the double strand is provided by the efficient π-stacking interaction [46]. Adding additional recognition sites to existing nucleotide receptor molecules can lead to multi-point recognition and enhanced selectivity/sensitivity for ATP chemosensors. In our ongoing research, we are interested in the exploration of the function of complementary base-pairing in ATP recognition as a possible way to enhance the selectivity. Since ATP has one adenine nucleobase, a simple uracil/thymine unit appended to a neutral chemosensor operating mainly through π-interaction could be a good model for investigation. We selected DMHF as the fluorophore and core scaffold because of its easy synthesis and ratiometric fluorescent nature. First, we examined the possible structure and the supramolecular interactions by quantum chemical calculations of our target compound, UHF (uracil-hydroxyflavone) and ATP (see Figure 1 for structures). The theoretical results indicated the possibility of base-pairing interactions, which prompted us towards the synthesis of UHF by click chemistry. Fluorescence spectroscopy revealed a selective complexation with ATP with an association constant of around 2∙10^4^ M^−1^ and a ratiometric response in the excitation spectrum.

## Results and Discussion

### Structure and calculations

Based on the detailed investigation of the supramolecular structure of the DEHF∙ATP (4’-diethylaminohydroxyflavone) complex (see below), we envisioned the uracil group to be appended on the A ring in close proximity to the nucleobase. In addition, some flexibility was required to obtain the proper conformation for the possible H-bonding. From the synthetic point of view the introduction of an uracil moiety can reasonably be accomplished at position 7 of DMHF with the appropriate C5*-*functionalized uracil derivative. The synthetic accessibility and the short spacer unit between the fluorophore and nucleobase were the main aspects in the design of UHF.

The influence of the side arm with the uracil group on the ability of UHF to act as an ATP sensor was investigated first by theoretical calculations, in which the structures and energies for the UHF∙ATP complex and – as a reference – for the DEHF∙ATP complex were computed.

Foremost, a molecular mechanical (MM) conformation analysis was performed for the individual molecules using the MMFF force field [47]. The stable conformers were optimized further at the density functional theory (DFT) level using the PBE functional [48–49] with D3BJ dispersion correction [50–51] and the 6-31G basis set [52]. Subsequently, the geometries obtained were utilized for generating the initial structures of the complexes, which were optimized with the same functional and basis set. To mimic the experimental conditions all the DFT calculations were performed using the polarized continuum model (PCM) [53] with water as solvent. The Marvin [54], ORCA [55], and MRCC [56] packages were used, respectively, for the MM, DFT, and local CC calculations.

**Figure 2:**
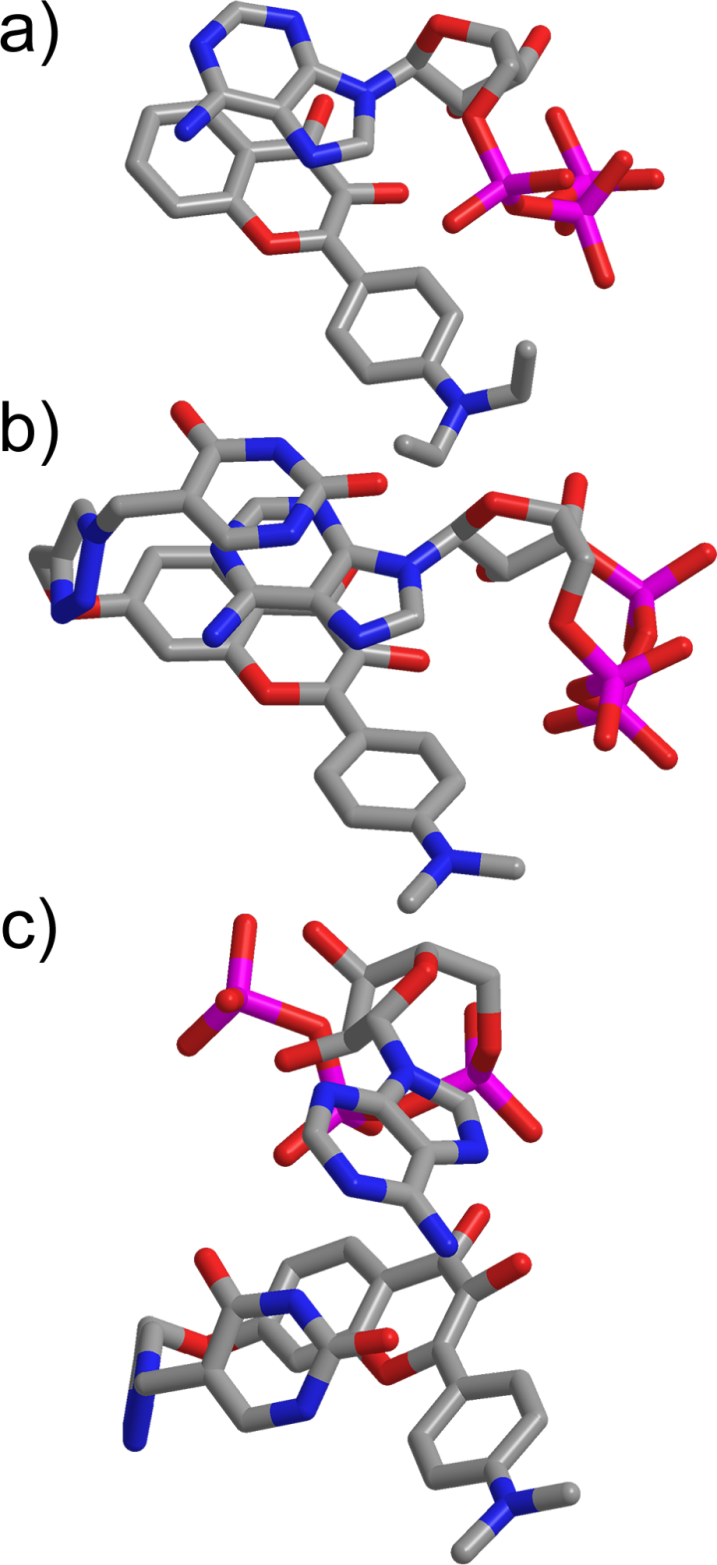
Optimized geometries for (a) DEHF∙ATP, (b) UHF∙ATP with the adenine of ATP “sandwiched” between the uracil and flavone units and (c) UHF∙ATP with hydrogen bonds between the uracil and the adenine moieties.

In the of case of the DEHF∙ATP complex one stable structure was found, while regarding the UHF∙ATP complex there were two. The optimized geometries are presented in Figure 2.

The investigation of the DEHF∙ATP 1:1 complex revealed a sandwich structure, which is held together by a π–π stacking- and an H-bond interaction. The triphosphate group is positioned near ring C of the DEHF and the π-stacking occurs between the adenine group and rings A and B of the flavone. The alignment of the flavones and ATP components is similar as in DMHF∙ATP, obtained by a computer aided simulation [34].

For the UHF∙ATP complex, two structurally rather different stable conformers were found in the calculations. In the first case (Figure 2b), the structure of the complex is similar to the DEHF∙ATP complex. However, an additional π-stacking interaction is formed between the adenine and the uracil groups creating a so-called “double-sandwich” structure. In the other case, the π–π interactions vanish and the complex is stabilized through the base-pair interactions (Figure 2c). Two H-bonds are formed between the adenine and the uracil groups, and two additional H-bonds between ring B of the flavone and the adenine group also stabilize the structure. To decide which structure is energetically more favorable, high accuracy local coupled-cluster (CC) calculations were performed. The complexation energies were computed using the local CC singles and doubles with perturbative triples [CCSD(T)] method [57] and the aug-cc-pVTZ basis set [58]. In the matter of the “double-sandwich” complex the complexation energy is 39 kcal/mol, while regarding the complex which is stabilized through the base-pair interactions 42 kcal/mol is obtained. The energies mentioned above are in accordance with the number and strength of the H-bonding and π-stacking interactions. Based on the numerical results, it can be stated that the formation of the base-pair interactions further stabilizes the complex, and this structure is energetically more favorable. These results prompted us towards the synthesis and evaluation of this promising molecule.

### Synthesis

**Scheme 1:**
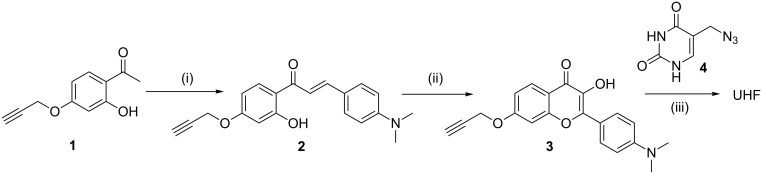
Synthesis of UHF. (i) 4-Dimethylaminobenzaldehyde, DMF, NaOMe, rt, 17 h, (ii) hydrogen peroxide, NaOH, ethanol, rt, 24 h, (iii) 5-azidomethyluracil, [Cu(MeCN)_4_]BF_4_, THF, rt, 24 h.

The synthesis of UHF is depicted in Scheme 1.

UHF was synthesized by the CuAAC (click) reaction of 7-propargyloxy-3-hydroxyflavone **3** and 5-azidomethyluracil (**4**) [59]. The hydroxyflavone was prepared according to the standard literature process for the preparation of these compounds [60]: the substituted hydroxyacetophenone **1** [61] was condensed to the corresponding chalcone using strongly basic conditions and reacted with alkaline hydrogen peroxide to obtain the clickable fluorophore. All new compounds were characterized by NMR and high-resolution mass spectrometry.

### Optical spectroscopy

**Figure 3:**
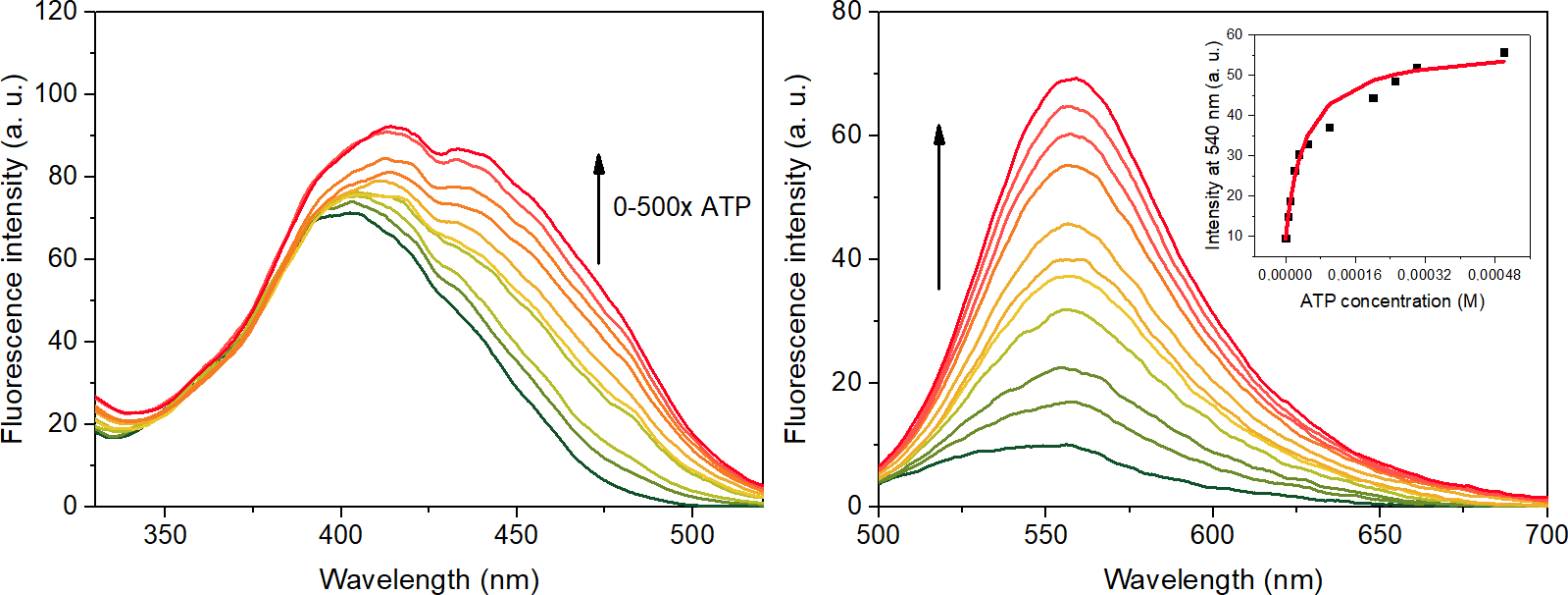
Variation of fluorescence spectra of UHF (1.0 μM) upon addition of increasing amounts of ATP in 0.02 M HEPES buffer which also contains 0.1 mM γ-cyclodextrin. Left: excitation spectra, detection wavelength: 540 nm; right: emission spectra, excitation wavelength: 470 nm. The inset shows the emission at 540 nm as a function of the ATP concentration, whereas the curve represents the result of a non-linear fitting to the spectra.

The solubility of the UHF probe was very poor in water which resulted in the decrease of fluorescence over time upon dilution from the stock solution in DMSO. The addition of γ-cyclodextrin as a solubilizer to the samples (HEPES buffer, pH 7.4) did not alter the spectra significantly, but provided a stable solution suitable for absorption and fluorescence titration experiments. The fluorescence spectra of UHF in the presence of ATP in different concentrations are shown in Figure 3.

Upon addition of ATP, a new band appears at 440 nm in the excitation spectra. This feature can be attributed to the specific intermolecular proton transfer from the hydroxy group of the flavone to the phosphate moiety of the ATP [34]. The fluorescence enhancement is remarkable upon excitation at 470 nm – a 7-fold increase can be observed using this excitation wavelength. No significant changes were recorded in the absorption spectra (Figure S15 in the Supporting Information File 1) using a 10 cm path length cuvette. The association constant was calculated for 1:1 and 1:2 complexes, a better fit was obtained for the unimolar complexation corroborating the theoretical results. The value was determined to be 2.3 ± 0.2∙10^4^ M^−1^ using non-linear curve fitting analysis from multiple titration experiments. The various flavones tested in [45] had association constants in the range of 0.3–3∙10^3^ M^−1^, considerably lower values. The effect of γ-cyclodextrin was examined in some preliminary experiments. Lowering the concentration to 0.05 mM resulted in unreliable spectra due to possible precipitation of the complex. A higher cyclodextrin concentration resulted in the same fluorescence response with an association constant of 5.7∙10^4^ M^−1^, however, the exact effect of various sized cyclodextrins on the complexation of flavones with ATP is currently unknown and will be the subject of an upcoming study by our group.

**Figure 4:**
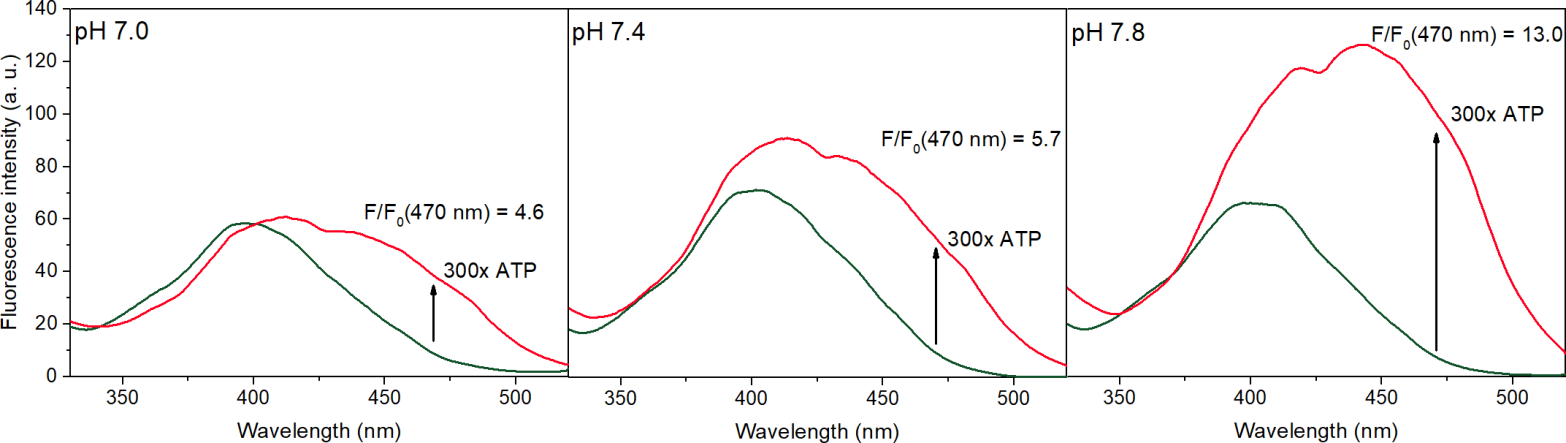
Excitation spectra of UHF (dark green line, 1 μM) and UHF + 300 equiv ATP (red line), measured at different pH values. The fluorescence enhancement values at 470 nm are also noted. The spectra were detected at 540 nm in 0.02 M HEPES buffer, in the presence of 0.1 mM γ-cyclodextrin.

To ascertain this method of signal transduction, the spectra of UHF and UHF∙ATP were recorded at different pH values (Figure 4). Since the p*K*_a_ value of the hydroxy group of the flavones is around 9 [54], no significant deprotonation should occur at pH 7.8. As can be seen in Figure 4, the excitation band of UHF around 400 nm is the same at each pH value. Upon addition of ATP, the most significant enhancement was recorded at pH 7.8, the pronounced band around 450 nm indicates the beneficial effect of this minor increase in the basicity of the media to the proton tranfer.

**Figure 5:**
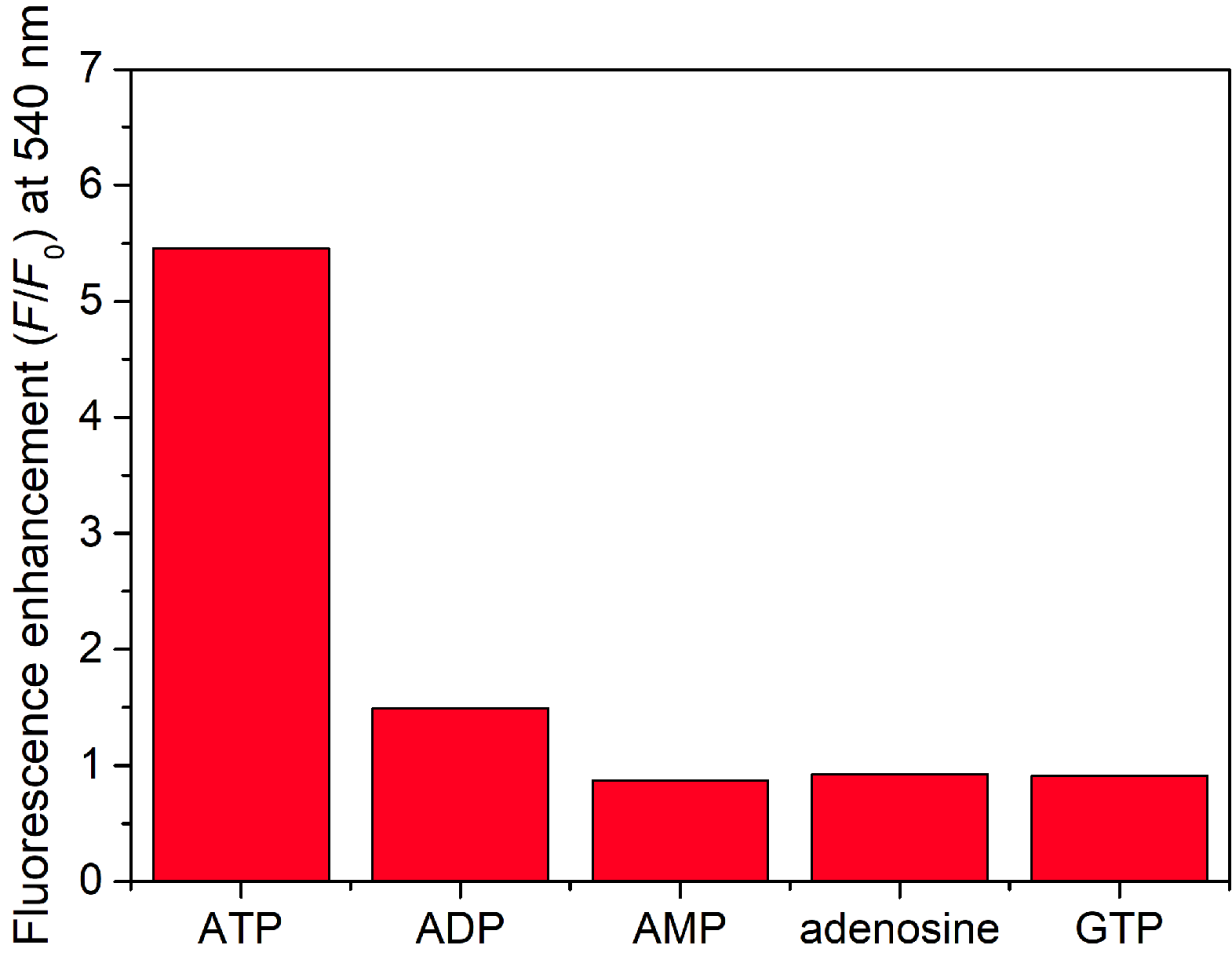
Fluorescence enhancement (*F*/*F*_0_) values of UHF (1.0 μM) upon addition of different nucleotides at 540 nm (excitation: 470 nm), in 0.02 M HEPES buffer in the presence of 0.1 mM γ-cyclodextrin. The analytes were added in 0.3 mM concentration.

The selectivity of the flavone probe has been investigated in a screening experiment; the results are summarized in Figure 5. As can be seen in Figure 5, among the different nucleotides, only ADP generated a slight fluorescence enhancement at 540 nm. According to [34], GTP and AMP caused a slight fluorescence enhancement in the case of DMHF; this was not detected in the case of UHF which might be the result of the complementary nucleobase attached to the flavone scaffold.

**Figure 6:**
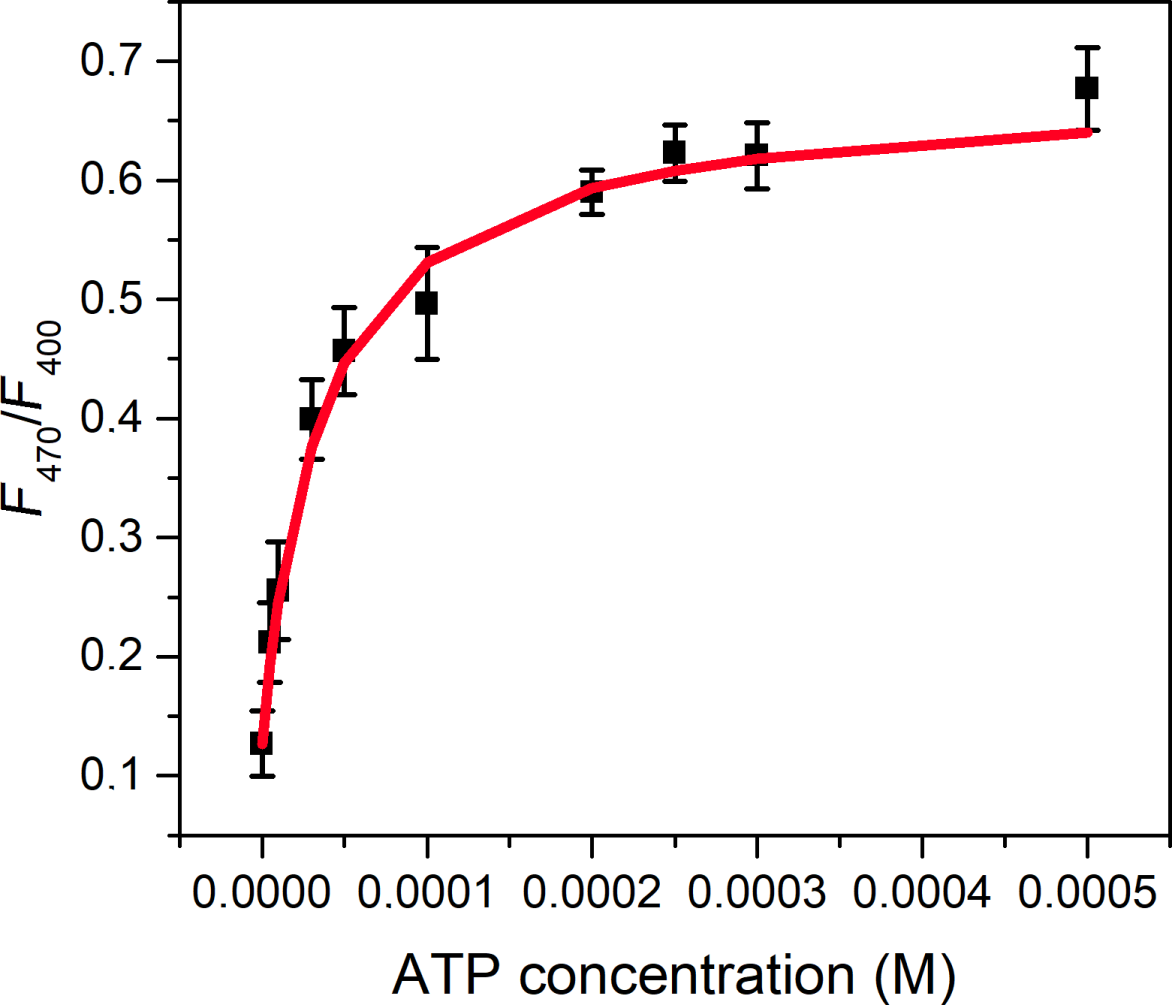
Ratio of the fluorescence intensities at 540 nm, the samples were excited at 470 and 400 nm. The red curve represents the result of a non-linear fitting.

Ratiometric fluorescence measurements received particular attention in the past decade due to their high sensitivity and reliability by their inherent self-calibration nature [62]. Most 3-hydroxyflavone-based probes exploit the ESIPT nature of these fluorophores to generate multiple emission bands [39,41–43]. In this case, however, a new fluorescence band appears in the excitation spectra due to an intermolecular proton transfer from the flavone to the phosphate chain of the nucleotide. Therefore, UHF can be applied as a ratiometric probe, setting different excitation wavelengths and measuring the fluorescence intensity at a selected wavelength. Figure 6 shows the intensity ratio *F*_470_/*F*_400_ (the subscript indicates two different excitation wavelengths) of UHF upon addition of ATP at 540 nm.

To the best of our knowledge, **3** is the first „clickable” hydroxyflavone. Considering the high interest in ratiometric fluorescent markers, we believe that this compound might be applicable in bioconjugate chemistry and related fields.

## Conclusion

In conclusion, we have designed a uracil-conjugated, 4’-amino-3-hydroxyflavone-based fluorescent probe (UHF) for the selective recognition of ATP. The theoretical results showed that the base-pairing interactions are feasible in the supramolecular structre of UHF∙ATP. The synthesized probe showed large fluorescence enhancement and a ratiometric response towards ATP with an association constant of 2.3∙10^4^ M^−1^. Excellent selectivity was observed with other nucleotides that might be the result of the beneficial effect of the complementary nucleobase.

## Experimental

### General

Solvents, reagents and starting materials were obtained from commercial suppliers and used without further purification. 5-Chloromethyluracil [63] was synthesized as described previously. The fluorescence spectra were measured on an Edinburgh Instruments FLSP 920 fluorescence spectrometer. The ^1^H NMR spectra were taken on a Bruker Avance DRX-500 or DRX-300 spectrometer with chemical shifts reported in ppm (TMS in in the case of CDCl_3_ and the residual DMSO in the case of DMSO-*d*_6_ was used as internal standard). The exact mass measurements were performed using a Q-TOF Premier mass spectrometer (Waters Corporation, 34 Maple St, Milford, MA, USA) using electrospray ionization in positive mode.

### Synthetic procedures

**1-(2-Hydroxy-4-(prop-2-yn-1-yloxy)phenyl)ethanone (1)** [61]**:** The propargyl compound **1** was synthesized as described with a modified purification method. 1-(2,4-dihydroxyphenyl)ethanone (6.00 g, 39.4 mmol, Sigma) was dissolved in acetone (90 mL). Potassium carbonate (6.54 g, 47.3 mmol, 1.2 equiv) and tetrabutylammonium bromide (2.54 g, 7.89 mmol, 0.2 equiv) were added and the mixture was cooled in an ice bath. Subsequently, propargyl bromide (80% in toluene, 4.83 mL, 43.3 mmol, 1.1 equiv) was added dropwise. The reaction mixture was stirred for 14 hours at ambient temperature. Upon completition, water was added (70 mL) and the pH was set to 5 using dilute hydrochloric acid. The mixture was extracted using ethyl acetate (3 × 70 mL), the organic phase was washed with water (3 × 50 mL) and brine (50 mL) and dried on MgSO_4_. The solvent was removed and the remaining off-white solid was recrystallized from 10 mL boiling ethanol to remove the unwanted dialkylated product and residual starting material. Yield: 4.21 g (56%) white crystals. ^1^H NMR (500 MHz, CDCl_3_) δ 12.70 (s, 1H, OH), 7.66 (m, 1H, 6-ArH), 6.51 (m, 2H, 3-,5-ArH), 4.72 (d, *J* = 2.5 Hz, 2H, OCH_2_), 2.57 (s, 3H, CH_3_), 2.56 (m, 1H, CH); ^13^C NMR (75 MHz, CDCl_3_) δ 202.84 (C=O), 165.12 (C_2-Ar-OH_), 163.94 (C_4-Ar-O_), 132.50 (CH_6-Ar_), 114.64 (C_1-Ar_), 107.99 (CH_3-Ar_), 102.20 (CH_2-Ar_), 77.58 (overlapping with CDCl_3_, C_alkyne_) 76.47 (CH_alkyne_), 56.06 (CH_2_), 26.42 (CH_3_).

**(*E*)-3-(4-(Dimethylamino)phenyl)-1-(2-hydroxy-4-(propargyloxy)phenyl)prop-2-en-1-one (2):** To a solution of **1** (1.50 g, 7.89 mmol) and 4-dimethylaminobenzaldehyde (1.18 g, 7.89 mmol, 1 equiv) in 15 mL anhydrous dimethylformamide was added sodium methoxide (1.53 g, 28.4 mmol, 3.6 equiv) and the resulting mixture was stirred at room temperature for 17 hours under an argon atmosphere. The deep red solution was poured into ice water (80 mL) and the pH was set to 5 using dilute hydrochloric acid. The mixture was extracted with ethyl acetate (3 × 30 mL), the organic phase was washed with water (3 × 30 mL) and brine (30 mL) and dried on MgSO_4_. The solvent was evaporated and the oily residue was crystallized from diethyl ether. The precipitate was collected by filtration and dried in vacuo to obtain 1.55 g (61%) of orange crystals. ^1^H NMR (500 MHz, CDCl_3_) δ 13.74 (s, 1H, OH), 7.90–7.82 (m, 2H, 6-ArH, CH=), 7.55 (d, *J* = 8.9 Hz, 2H, 2’-ArH), 7.36 (d, *J* = 15.2 Hz, 1H, CH=), 6.69 (d, *J* = 8.9 Hz, 2H, 3’-ArH), 6.57–6.48 (m, 2H, 5-ArH, 3-ArH), 4.73 (d, *J* = 2.4 Hz, 2H, OCH_2_), 3.05 (s, 6H, CH_3_), 2.57 (t, *J* = 2.4 Hz, 1H, alkyne); ^13^C NMR (126 MHz, CDCl_3_) δ 192.13 (C=O), 166.32 (C_2Ar-OH_), 163.52 (C_4Ar-O_), 152.37 (C_4’-Ar-N_), 145.85 (CH_double bond, Ar-C_), 131.15 (CH_6-Ar_), 130.81 (2CH_2’-Ar_), 122.64 (C_Ar or double bond_), 115.15 (C_Ar or double bond_), 114.61 (C_Ar or double bond_), 111.97 (2C_3’-Ar_), 107.67 (CH_3-Ar_), 102.39 (CH_2-Ar_), 77.80 (C_alkyne_), 76.35 (CH_alkyne_), 56.05 (CH_2_), 40.24 (CH_3_); HRMS calcd. for [M + H^+^]: 322.1443; found: 322.1443.

**2-(4-(Dimethylamino)phenyl)-3-hydroxy-7-propargyloxy-4*H*-chromen-4-one (3):** Chalcone **2** (500 mg, 1.56 mmol) was dissolved in ethanol (25 mL) and sodium hydroxide (700 mg, 17.6 mmol, 11 equiv), dissolved in water (12.5 mL), was added. To the deep red solution was added 0.75 mL 30% hydrogen peroxide and the mixture was stirred at room temperature. After 24 hours, the yellow solution was poured into ice water and pH 5 was set by concentrated acetic acid. The pure product precipitated as yellow crystals, filtered, washed with water and dried in vacuo to yield 401 mg (77%) product. ^1^H NMR (500 MHz, DMSO-*d*_6_) δ 9.09 (s, 1H, OH), 8.10 (d, *J* = 8.8 Hz, 2H, 2’-ArH), 7.99 (d, *J* = 8.8 Hz, 1H, 5-ArH), 7.30 (d, *J* = 2.4 Hz, 1H, 8-ArH), 7.06 (dd, *J* = 8.8, 2.4 Hz, 1H, 6-ArH), 6.85 (d, *J* = 8.8 Hz, 2H, 3’-ArH), 4.99 (d, *J* = 2.5 Hz, 2H, OCH_2_), 3.69 (t, *J* = 2.4 Hz, 1H, alkyne), 3.02 (s, 6H, CH_3_); ^13^C NMR (75 MHz, CDCl_3_) δ 171.47 (C=O), 160.96 (C_Ar_-O), 155.78 (C_Ar_-O), 150.86 (C_4’-Ar_-N), 146.27 (C_Ar_-O), 136.89 (C-OH), 128.66 (2CH_2’-Ar_), 126.08 (CH_Ar_), 118.01 (C_Ar_), 115.75 (C_Ar_), 114.38 (CH_Ar_), 111.37 (2CH_3’-Ar_), 101.47 (CH_Ar_), 78.99 (C_alkyne_), 78.52 (CH_alkyne_), 56.18 (CH_2_), 39.64 (CH_3_); HRMS calcd. for [M + H^+^]: 336.1236; found: 336.1232.

**5-Azidomethyluracil (4)** [59]**:** The azido compound was synthesized as previously described. To a solution of 5-chloromethyluracil [63] (1.00 g, 6.23 mmol) in dimethylformamide (24 mL), sodium azide (0.81 g, 12.5 mmol, 2 equiv) was added. The mixture was stirred at ambient temperature for 1 h, then poured to 50 mL of water. The resulting solution was extracted with ethyl acetate (5 × 30 mL), the organic phase was washed with water (40 mL) and dried on MgSO_4_. After evaporation, the oily residue was crystallized from diethyl ether, filtered and dried to give 0.46 g (44%) product as white crystals. ^1^H NMR (500 MHz, DMSO-*d*_6_) δ 11.29 (s, 1H, 3-uracil NH), 10.99 (s, 1H, 1-uracil NH), 7.64 (s, 1H, 6-uracil CH), 4.02 (s, 2H, CH_2_); ^13^C NMR (75 MHz, DMSO-*d*_6_) δ 163.97 (C=O_6-uracil_), 151.23, 141.94 (CH_4-uracil_), 106.72 (C_5-uracil_), 46.56 (CH_2_).

**Uracil-hydroxyflavone probe (UHF):** The click reaction of **3** and **4** was performed as follows. Propargyl derivative **3** (200 mg, 0.596 mmol) and azide compound **4** (100 mg, 0.596 mmol, 1 equiv) was dissolved in tetrahydrofuran (25 mL), and TBTA [64] (32 mg, 0.1 equiv) and [Cu(MeCN)_4_]BF_4_ (14 mg, 0.075 equiv) were added. The reaction mixture was stirred for 24 h, and the product precipitated from the solution. The precipitate was filtered, washed with THF thoroughly and dried to yield 264 mg (88%) product as a yellow solid. ^1^H NMR (500 MHz, DMSO-*d*_6_) δ 11.16 (s, 1H, 3-uracil NH), 10.94 (br s, 1H, 1-uracil NH), 8.79 (s, 1H, OH), 8.21 (s, 1H, ArH, triazole), 8.10 (d, *J* = 8.5 Hz, 2H, 2’-ArH, aniline), 7.97 (d, *J* = 8,8 Hz, 1H, ArH, 5-chromone), 7.72 (s 1H, 6-uracil), 7.39 (s, 1H, ArH, 8-chromone), 7.07 (d, *J* = 9,3 Hz, 1H, ArH, 6-chromone), 6.85 (d, *J* = 8.6 Hz, 2H, 3’-ArH, aniline), 5.30 (s, 2H, CH_2_), 5.19 (s, 2H, CH_2_), 3.02 (s, 6H, CH_3_); ^13^C NMR (126 MHz, DMSO) δ 171.46 (C=O_chromone_), 163.64 (C=O_6-uracill_), 161.90 (C_Ar-O_), 155.98 (C_Ar-O_), 151.16 (C=O_2-uracil_), 150.85 (C_4’-Ar, aniline_), 146.17 (C_Ar-O_), 142.98 (CH_Ar, triazole_), 141.70 (CH_4-uracil_), 136.82 (C-OH_chromone_), 128.64 (2CH_2’-Ar, aniline_), 125.96 (CH_Ar, chromone_), 124.83 (CH_Ar, triazole_), 118.06 (C_Ar, chromone_), 115.42 (C_Ar, chromone_), 114.46 (CH_Ar, chromone_), 111.37 (2CH_3’-Ar, aniline_), 106.18 (C_5-uracil_), 101.15 (CH_Ar, chromone_), 61.75 (CH_2_-O), 45.90 (CH_2_-N), 40.02 (CH_3_); HRMS calcd. for [M + H^+^]: 503.1679; found: 503.1675.

### Fluorescence measurements

All the spectroscopic experiments were carried out at 25 °C. In all experiments, 0.02 M HEPES was used as buffer solution. Since the solubility of UHF in pure water is negligible, a stock solution of 1.0 mM was prepared in DMSO which was diluted with the buffered solution of γ-cyclodextrin (0.1 mM) and the analyte. The DMSO content in these samples was well below 1%. Each spectrum was measured after reaching the equilibrium (5 minutes), and by using γ-cyclodextrin, it was stable over a longer period of time. Since the spectra of UHF and UHF∙ATP did not change upon addition of γ-cyclodextrin in pure HEPES buffer (measurement performed before precipitation), it is unlikely that they form any type of inclusion complexes disturbing the recognition of ATP.

### Association constant determination

The association constant has been obtained from the emission spectra using standard methods for non-linear curve fitting [65]. The best fit was obtained using 1:1 stoichiometry which confirmed our model of complexation [66].

## Supporting Information

File 1 NMR spectra and additional figures.
