# Supplementary material for: An uracil-linked hydroxyflavone probe for the recognition of ATP
Source: Beilstein J Org Chem. 2018 Apr 3;14:747–55. doi: 10.3762/bjoc.14.63 (PMC5905274; doi:10.3762/bjoc.14.63)
Supplement: File 1 — NMR spectra and additional figures. [file Beilstein_J_Org_Chem-14-747-s001.pdf]

Supporting Information

for

An uracil-linked hydroxyflavone probe for the recognition of ATP

Márton Bojtár[1], Péter Zoltán Janzsó-Berend[1], Dávid Mester[2], Dóra Hessz[3], Mihály Kállay[2], Miklós Kubinyi[3,4] and István Bitter*[1]

Address: [1]Department of Organic Chemistry and Technology, Budapest University of Technology and Economics, 1521 Budapest, Hungary, [2]MTA-BME Lendület Quantum Chemistry Research Group, Department of Physical Chemistry and Materials Science, Budapest University of Technology and Economics, 1521 Budapest, Hungary, [3]Institute of Materials and Environmental Chemistry, Research Center for Natural Sciences, Hungarian Academy of Sciences, P. O. Box 286, 1519 Budapest, Hungary and [4]Department of Physical Chemistry and Materials Science, Budapest University of Technology and Economics, 1521 Budapest, Hungary

*Corresponding author

Email: István Bitter - bitter@oct.bme.hu

NMR spectra and additional figures

1. NMR spectra of compounds

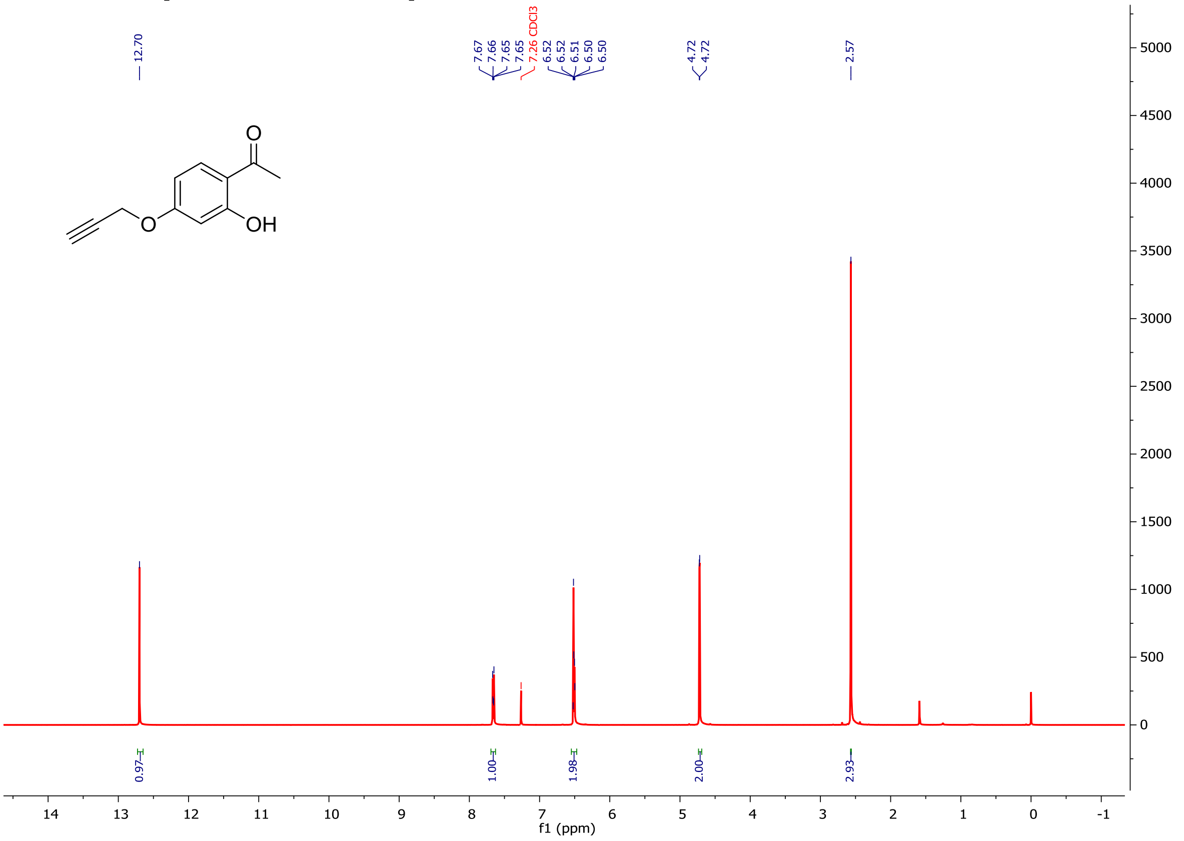

Figure S1: ^{1}H NMR spectrum of **1** ($CDCl_3$, 500 MHz, 298 K).

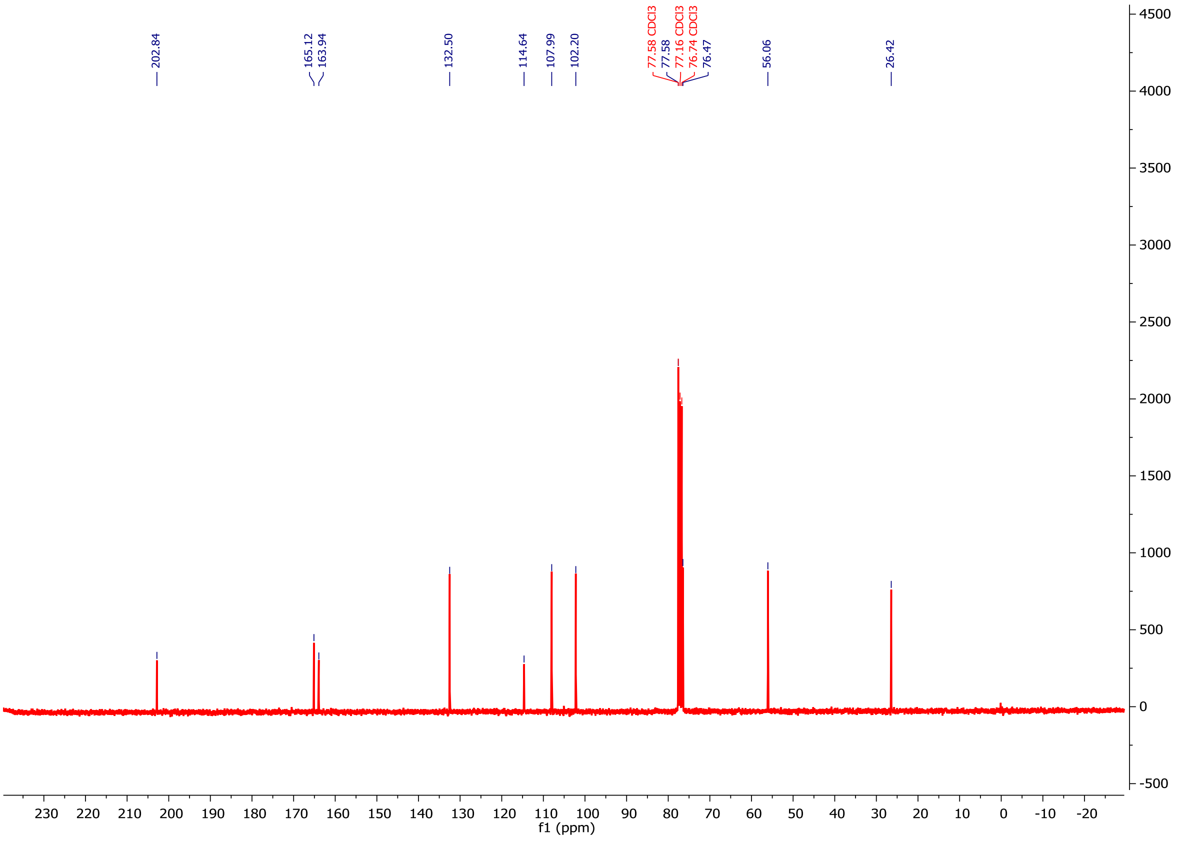

Figure S2: ^{13}C NMR spectrum of **1** ($CDCl_3$, 75 MHz, 298 K).

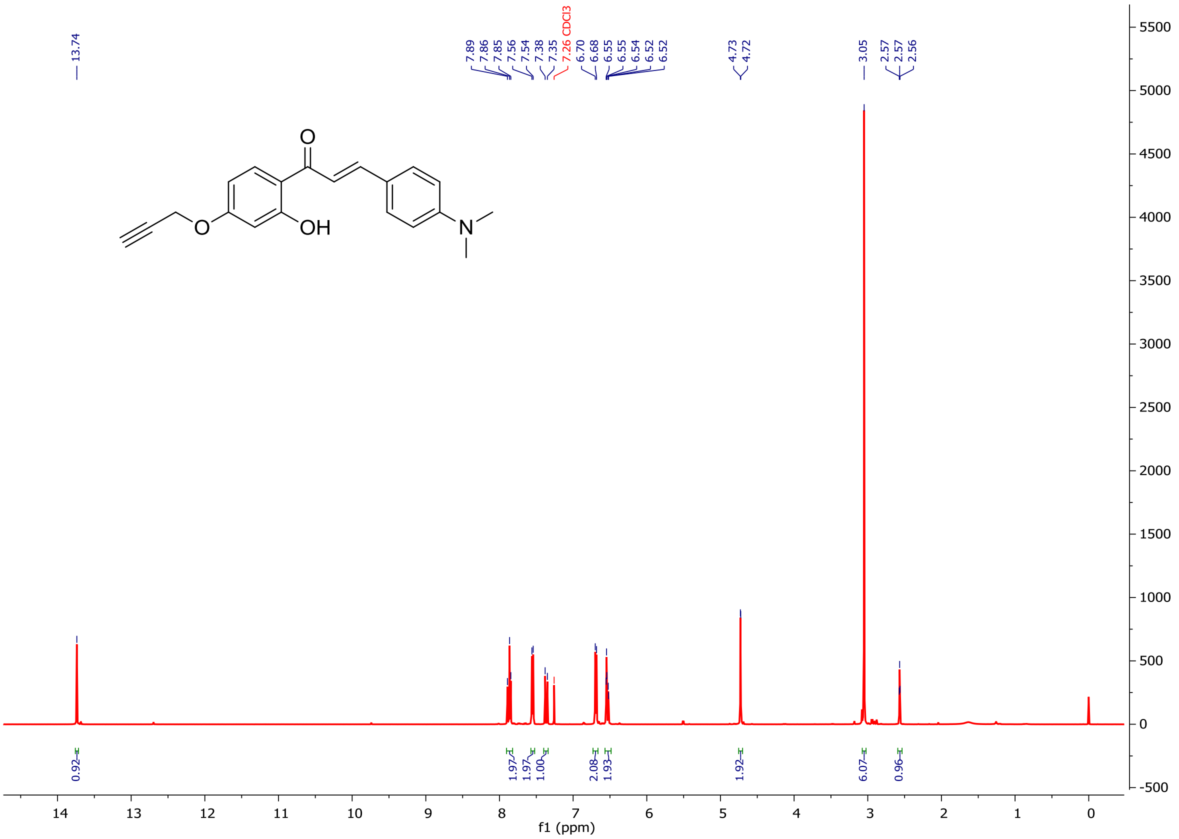

Figure S3: ^{1}H NMR spectrum of **2** ($CDCl_3$, 500 MHz, 298 K).

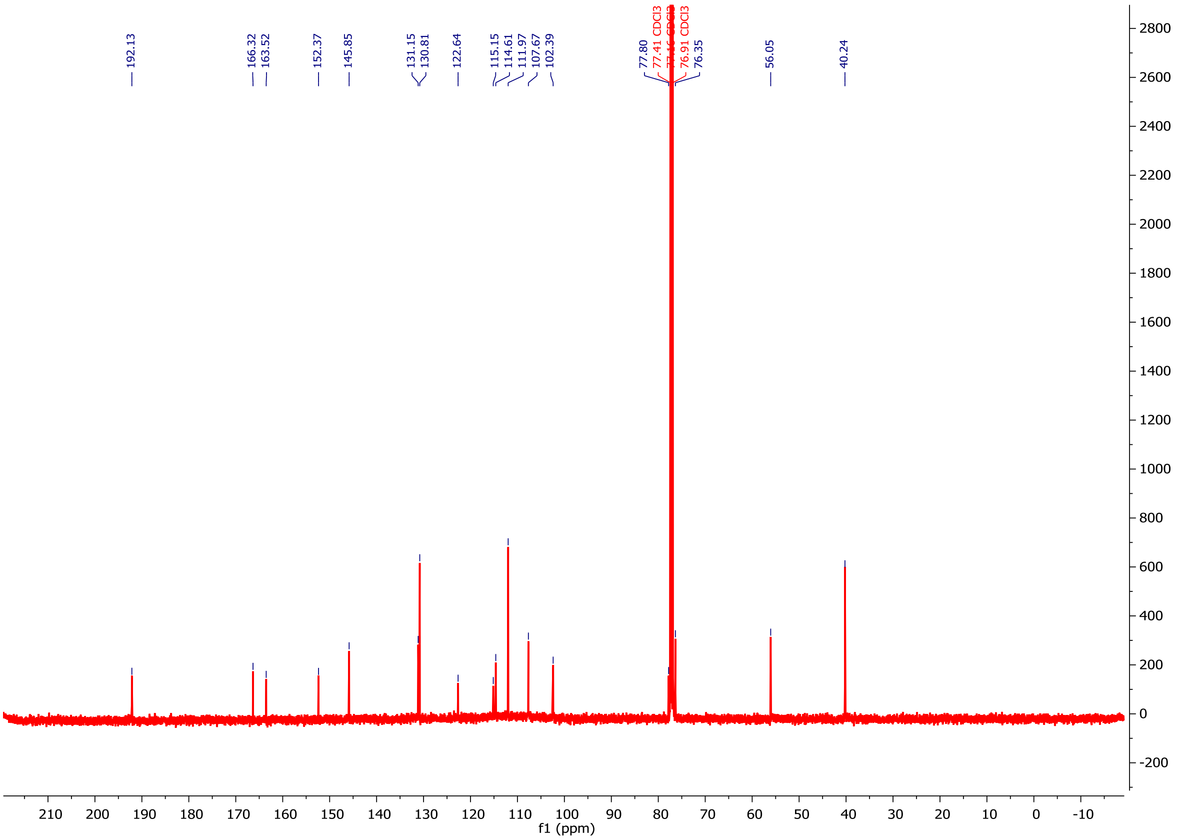

Figure S4: ^{13}C NMR spectrum of **2** ($CDCl_3$, 126 MHz, 298 K).

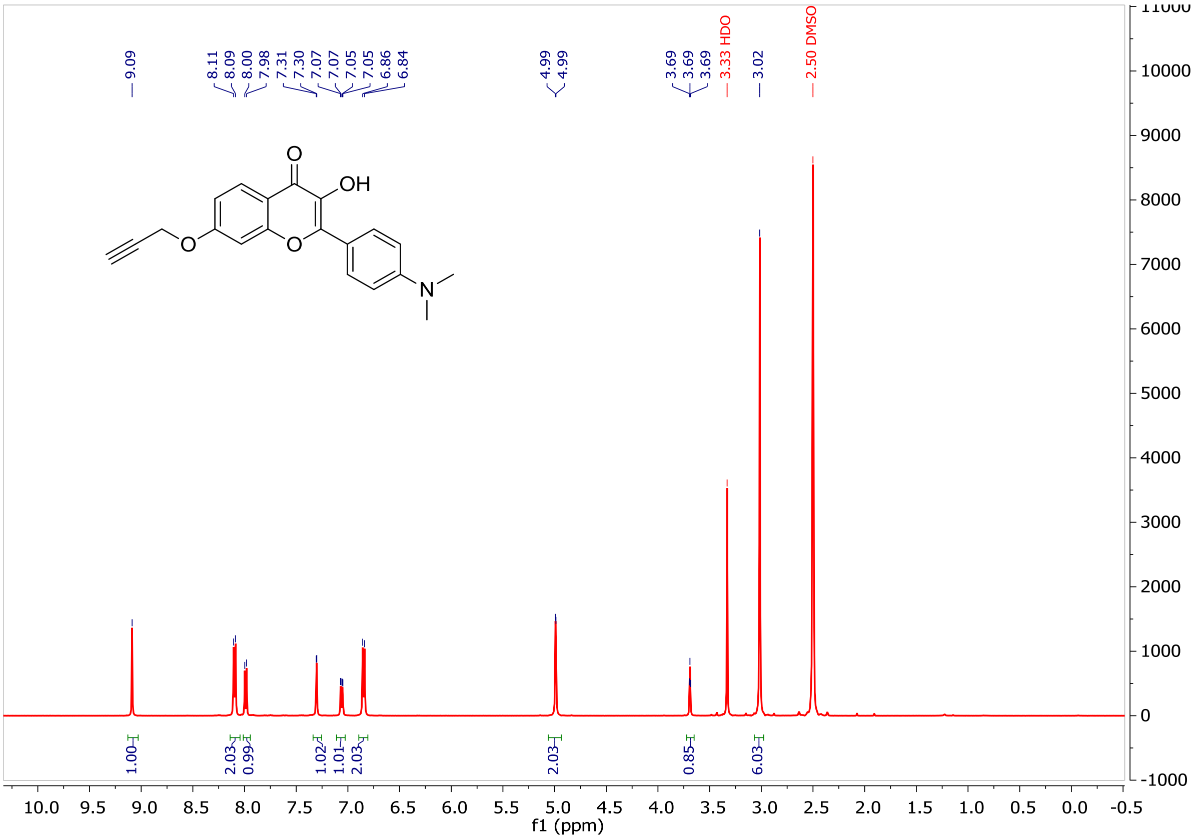

Figure S5: ^{1}H NMR spectrum of **3** (DMSO, 500 MHz, 298 K).

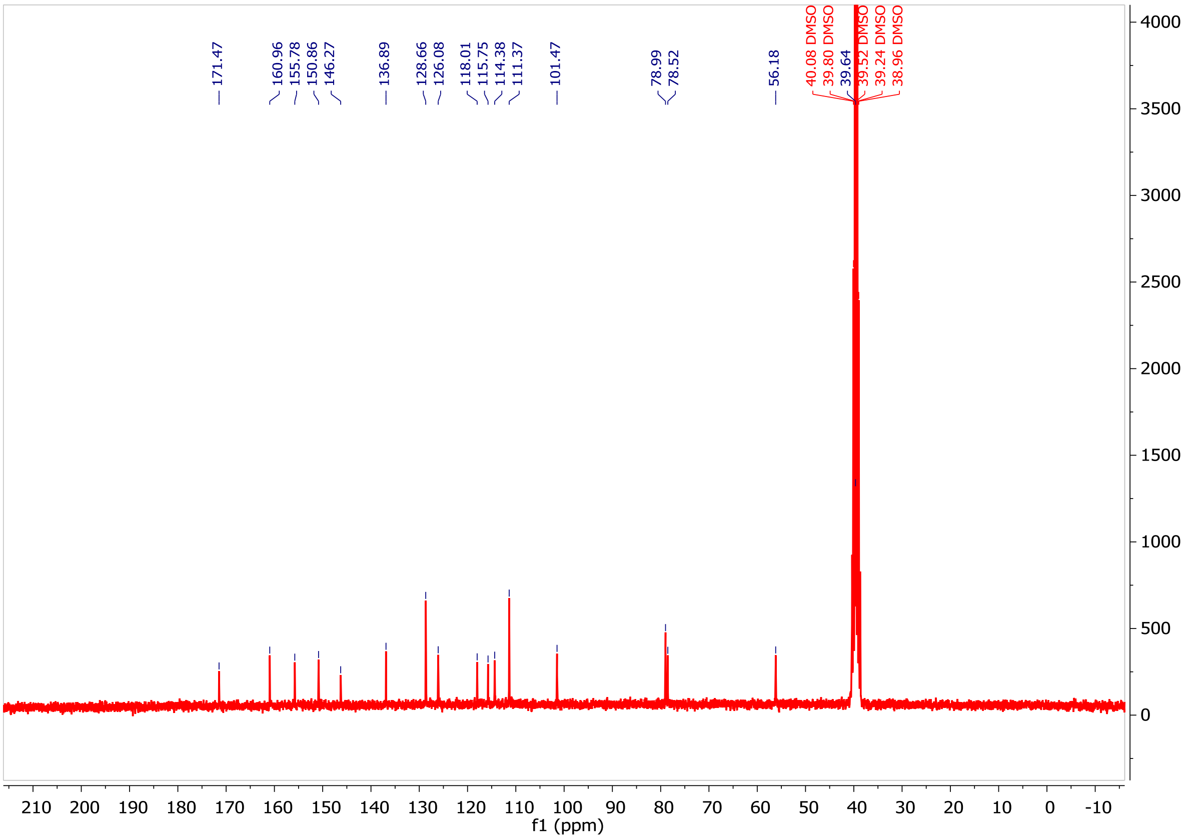

Figure S6: ^{13}C NMR spectrum of **3** (DMSO, 75 MHz, 298 K).

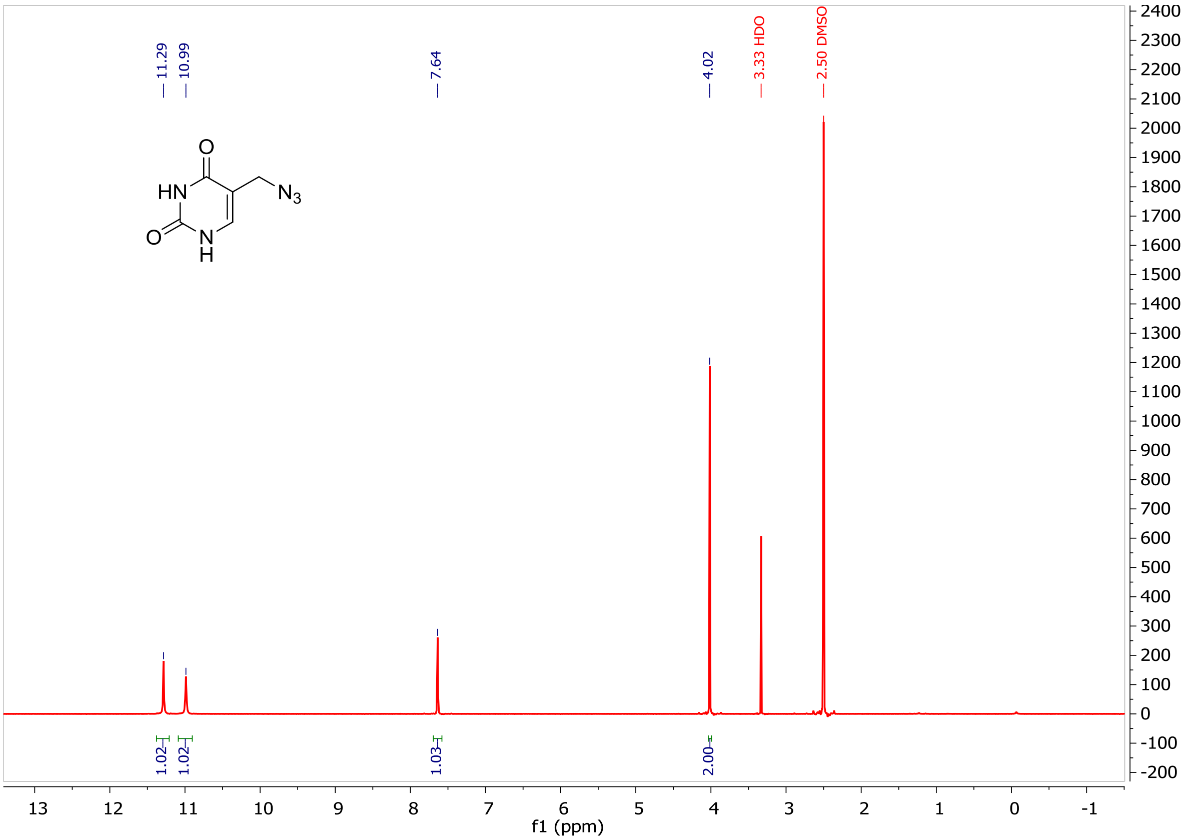

Figure S7: ^{1}H NMR spectrum of **4** (DMSO, 500 MHz, 298 K).

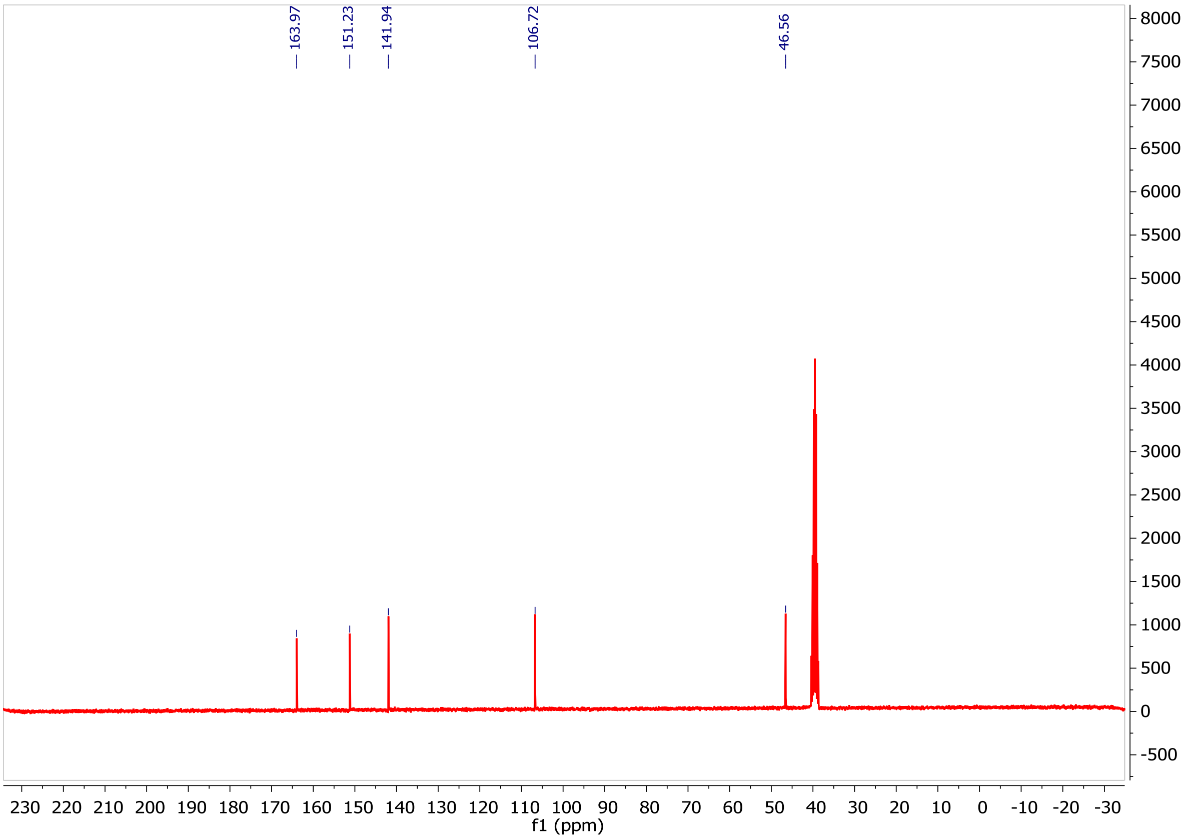

Figure S8: ^{13}C NMR spectrum of **4** (DMSO, 75 MHz, 298 K).

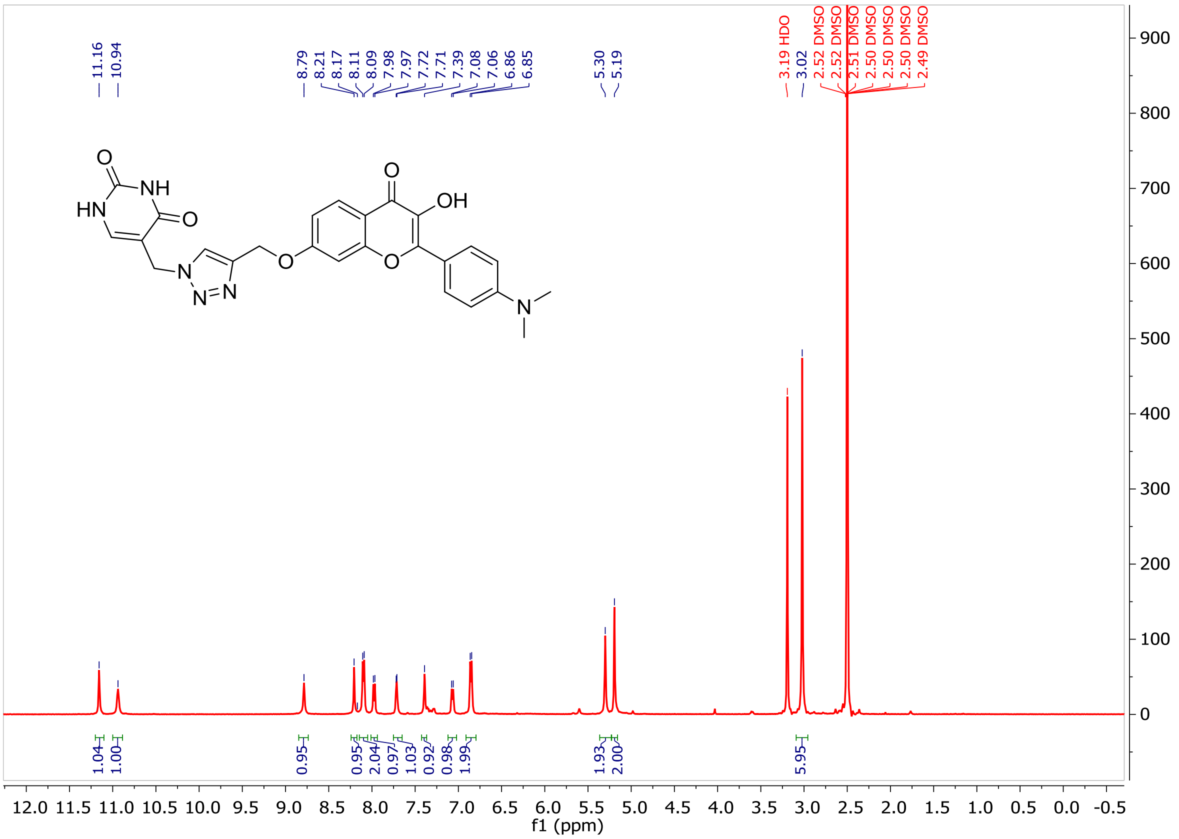

Figure S9: ^{1}H NMR spectrum of **UHF** (DMSO, 500 MHz, 298 K).

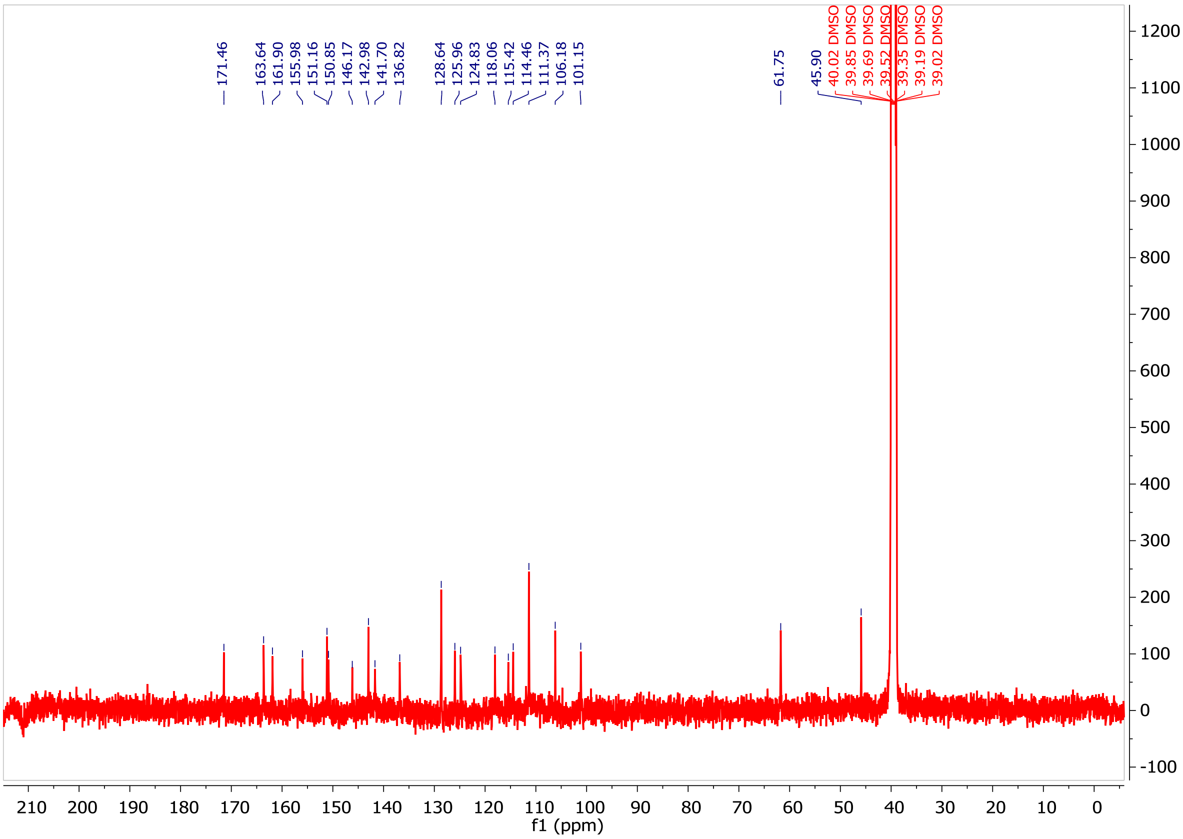

Figure S10: ^{13}C NMR spectrum of **UHF** (DMSO, 126 MHz, 298 K).

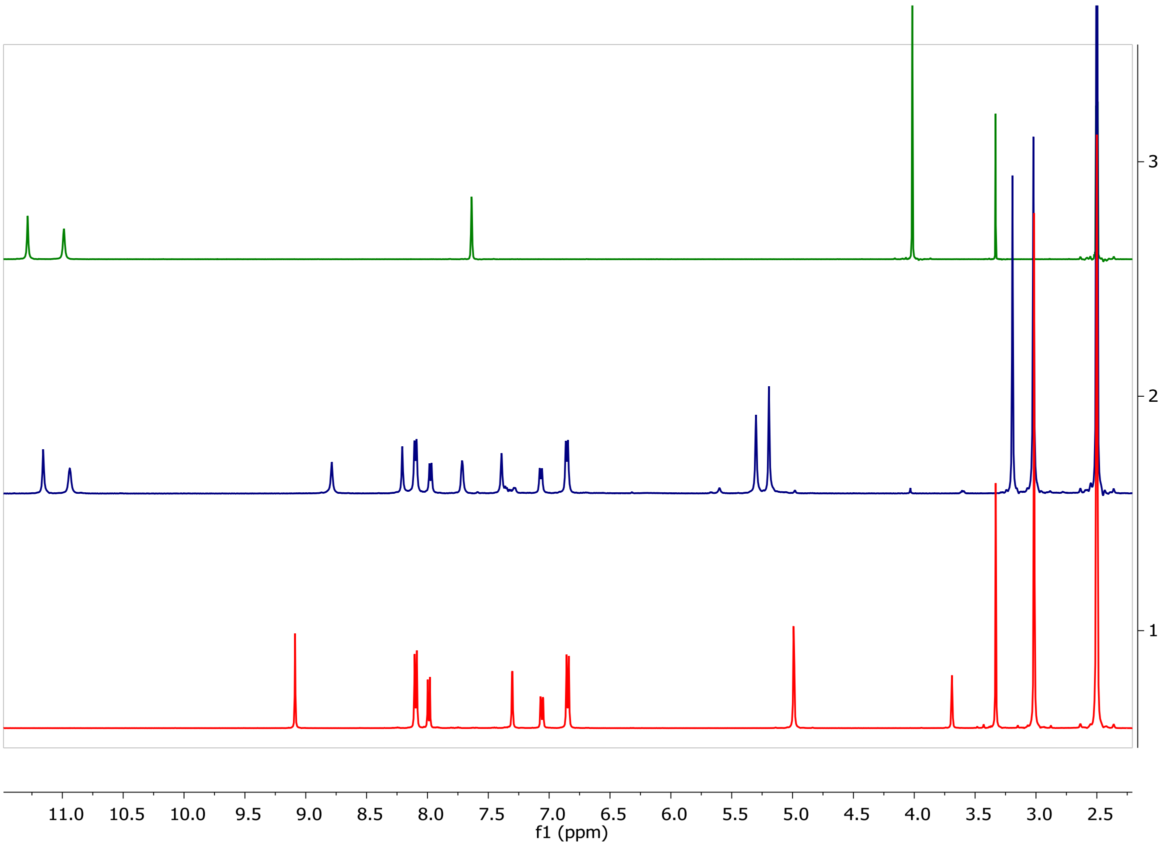

Figure S11: Stacked ^{1}H NMR spectra of (top) **4** (middle) UHF (bottom) **3** (DMSO, 500 MHz, 298 K) for the assignment of the protons before and after the click reaction.

2. HRMS data of compounds

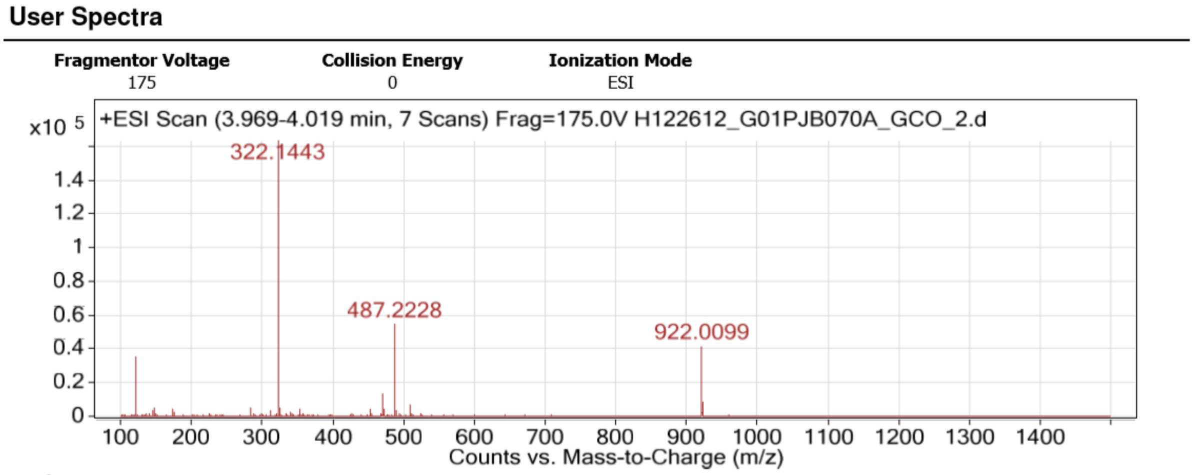

Figure S12: HRMS spectrum of **2**. Calculated mass for $[M + H]^{+}$: 322.1443

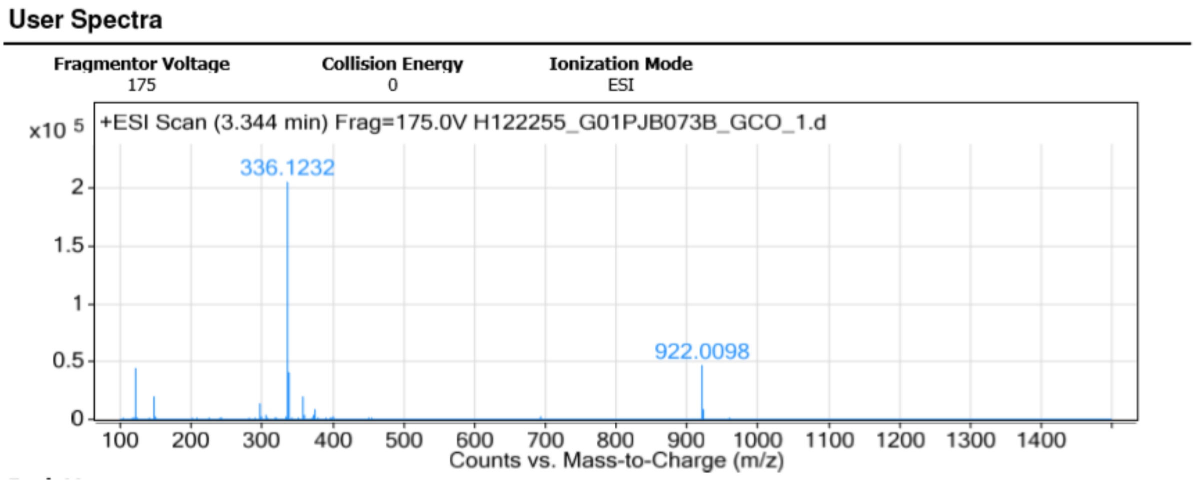

Figure S13: HRMS spectrum of **3**. Calculated mass for $[M + H]^+$: 336.1236

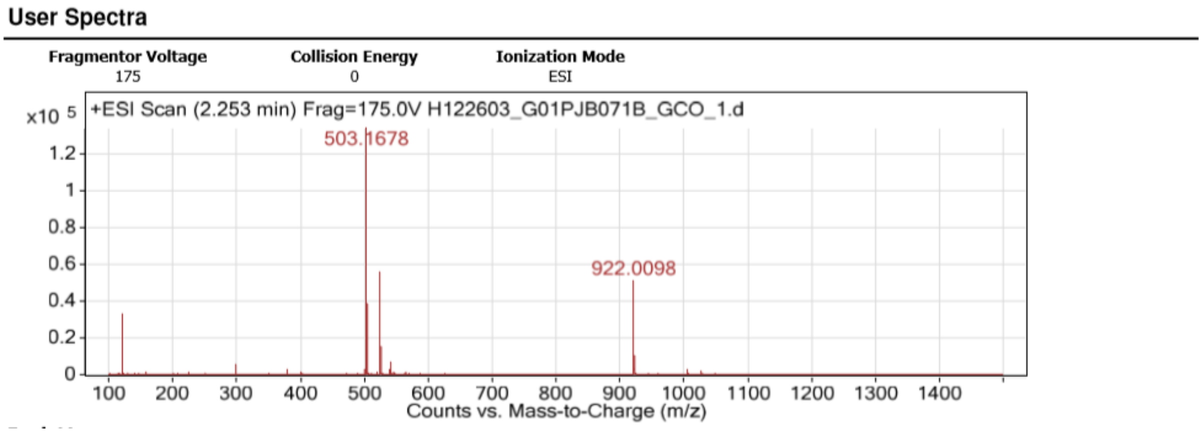

Figure S14: HRMS spectrum of **UHF**. Calculated mass for $[M + H]^+$: 503.1679

Note: HRMS data for **1** and **4** can be found in the literature ([1,2]).

3. Absorption spectra

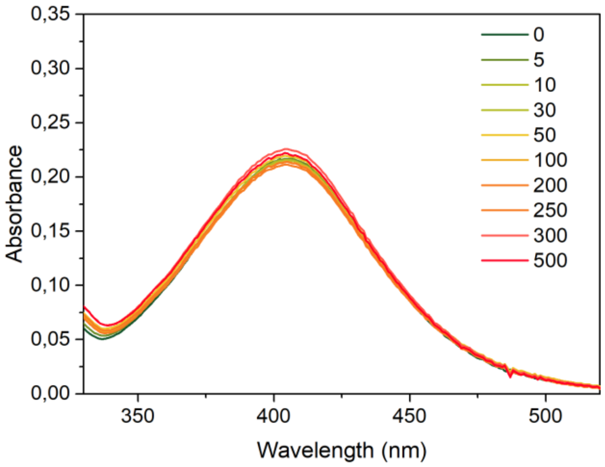

Figure S15: UV–vis spectra of UHF (1.0 μM) upon addition of increasing amount of ATP in 0.02 M HEPES which also contains 0.1 mM γ-cyclodextrin using a cuvette with a path length of 10 cm. The ATP equivalents to the probe are shown on the graph.

References

1. Anand, N.; Singh, P.; Sharma, A.; Tiwari, S.; Singh, V.; Singh, D. K.; Srivastava, K. K.; Singh, B. N.; Tripathi, R. P. *Bioorg. Med. Chem.* **2012,** *20,* 5150–5163. doi:10.1016/j.bmc.2012.07.009
2. Nguyen, K.; Fazio, M.; Kubota, M.; Nainar, S.; Feng, C.; Li, X.; Atwood, S. X.; Bredy, T. W.; Spitale, R. C. *J. Am. Chem. Soc.* **2017,** *139,* 2148–2151. doi:10.1021/jacs.6b11401
